# Differences in Body Composition across Police Occupations and Moderation Effects of Leisure Time Physical Activity

**DOI:** 10.3390/ijerph17186825

**Published:** 2020-09-18

**Authors:** Filip Kukić, Katie M. Heinrich, Nenad Koropanovski, Walker S. C. Poston, Aleksandar Čvorović, J. Jay Dawes, Robin Orr, Milivoj Dopsaj

**Affiliations:** 1 Police Sports Education Centre, Abu Dhabi Police, 253 Abu Dhabi, UAE; cvorovic77@yahoo.com; 2 Department of Kinesiology, Kansas State University, Manhattan, KS 66506, USA; kmhphd@ksu.edu; 3 Department of Criminalistics, University of Criminal Investigation and Police Studies, 11080 Belgrade, Serbia; nenad.koropanovski@kpu.edu.rs; 4 NDRI-USA, New York, NY 10001, USA; poston@ndri-usa.org; 5 School of Kinesiology, Applied Health and Recreation, Oklahoma State University, Stillwater, OK 74074, USA; jay.dawes@okstate.edu; 6 Tactical Research Unit, Bond University, Robina, QLD 4226, Australia; rorr@bond.edu.au; 7 Faculty of Sport and Physical Education, University of Belgrade, 11000 Belgrade, Serbia; milivoj.dopsaj@gmail.com; 8 Institute of Sport, Tourism and Service, South Ural State University, 454080 Chelyabinsk, Russia

**Keywords:** skeletal muscle mass, body fat, occupational health, tactical athletes, law enforcement

## Abstract

This study investigated differences in the body composition of police officers from different occupational groups and the moderation effects of leisure time physical activity (LTPA). A sample of 237 police officers (special anti-terrorist unit, gendarmerie, firefighters, and general duties) participated in the study. Body mass index (BMI), percent of body fat (%BF), percent of skeletal muscle mass (%SMM), and index of hypokinesia (IH) were assessed using a multichannel bioelectric impedance analyzer and officers reported the amount of LTPA using an international physical activity questionnaire. The sample was divided in three groups relative to LTPA, sedentary (0–149 min/week), moderately active (150–299 min/week), and very active (300+ min/week). Multiple analysis of variance (4 × 3) was used to analyze if occupational groups differed based solely on occupation, if officers from the same occupation differed in muscularity and fatness based on LTPA, and if any differences in body composition occurred between officers from different occupations with the same level of LTPA. Police officers from more physically demanding occupations demonstrated lower %BF and IH, while having higher %SMM. This was also observed among the officers of the same occupation who reported a higher LTPA, while officers who reported very high LTPA had similar body composition.

## 1. Introduction

The tasks performed differ across police occupations, some of which are physically demanding (i.e., pursuing and engaging in physical confrontations with violent offenders), while others are more sedentary (i.e., administrative work) [1,2,3]. To perform physically demanding tasks effectively and with a reduced risk of injury, performance-related and health-related physical fitness [4,5,6] is very important for police officers [7,8,9,10]. Assessments of physical performance in police officers have often been associated with measures of body fat mass and skeletal muscle mass [5,8,11].

Body fatness, indicated through measures such as percent body fat (%BF), waist circumference, and body mass index (BMI—body mass (kg) divided by body height (m^2^)), increases due to lack of physical activity, sedentary work, and shift work in police officers [12,13,14,15,16]. In contrast, sufficient physical activity, planned exercise, and healthy nutrition are related to reductions in body fat mass and %BF and increases in percent of skeletal muscle mass (%SMM) and skeletal muscle mass index (SMMI) [6,16,17,18]. Therefore, assessment of body composition can be a useful tool to understand and monitor the effects that physical activity, lifestyle, and dietary habits have on police officers.

Evidence shows that age plays a role in body composition changes for police officers. Sorrensen et al. [12] found negative changes in BMI (average increase of 2.1 kg/m^2^, *p* < 0.001), and body mass ((BM), average increase of 7 kg, *p* < 0.001 or 0.5 kg/year of service) in Finnish police officers after 15 years of service. These health-related factors may contribute to the significantly shorter life expectancy for police officers compared to the general population [19]. In contrast, exercise and nutrition can positively influence body composition among police officers. Demling and DeSanti [18] found that resistance training followed by an increased protein diet led to a significant (*p* < 0.05) decrease of approximately 5.60% in %BF and an increase of approximately 3% in percent of lean mass. Furthermore, significant (*p* < 0.001) reductions in BM (7.50%), BMI (7.48%), waist circumference (8.26%) and waist-to-height ratio (9.26%) occurred after a 12-week exercise and diet programme in overweight police trainees [17]. Vuković et al. [16] investigated the effects of leisure time physical activity (LTPA) on indicators of body fatness and muscularity of police officers and found that those who were very active had a lower %BF and body fat mass index (BFMI) and a higher percent of skeletal muscle mass (%SMM) and SMMI. Of note, a physical activity frequency of 3–5 times/week was positively correlated with indicators of muscularity status (%SMM, SMMI), while physical activity volumes of 150–300 min/week and 300+ min/week were positively correlated with reduced fatness (%BF, BFMI).

Police officers’ daily duties influence their body composition as well as the frequency and volume of their physical activity at work and during leisure time. The sedentary activities an officer is required to do on a daily basis (e.g., deskwork) can leave little available time for physical activity and/or exercise due to operational work overload [15,20,21]. On the other hand, police tactical personnel, such as special weapons and tactics, antiterrorist units and, in some countries, firefighters, are normally selected based on their level of physical fitness [3]. Furthermore, their jobs entail additional skills and physical preparedness training [22,23]. Moreover, some agencies allow these specialist tactical personnel to complete strength and conditioning programs during working hours to maintain or improve physical fitness [22,23,24]. The importance of this on-duty physical training time is reflected in differences in the fitness and body composition characteristics of these personnel. Lower BMI and body fat mass (BFM) in full-time (mandatory training during work time with a certified strength and conditioning coach) compared to part-time (who train on their own time) specialist tactical personnel serves as an example [25]. However, limited research has been conducted to investigate the differences in body composition of various police occupations and even less has been performed in regard to the relationship between police occupations and differences in LTPA levels.

Consideration of these factors in police agencies is important from the perspective of occupational health as it could help to determine how officers’ body composition and physical activity levels differ between police occupations and whether LTPA could be a significant moderator of body composition. Therefore, the aims of this study were to investigate differences in body composition and levels of LTPA between various police occupations, and whether LTPA, more so than occupation, moderates the relationship between occupation and body composition. We hypothesized that police officers from more physically demanding occupations will have lower levels of body fat, greater %SMM, and that they will report higher levels of physical activity. We also hypothesized that LTPA could be a significant moderator of body composition.

## 2. Materials and Methods

### 2.1. Participants

The sample consisted of 237 male police officers recruited randomly from four different occupations: Special Anti-Terrorist Unit ((SAU), *n* = 54), Gendarmerie (*n* = 55), firefighters (*n* = 68), and general police officers (*n* = 60). The main characteristics of groups were: SAU (age = 36.72 ± 4.96 years, body height (BH) = 181.63 ± 5.30 cm, BM = 89.13 ± 9.57 kg, and BMI = 26.99 ± 2.17 kg/m^2^); Gendarmerie (age = 35.65 ± 4.88 years, BH = 181.79 ± 7.05 cm, BM = 92.58 ± 15.84 kg, and BMI = 27.92 ± 3.74 kg/m^2^); firefighters (age = 34.44 ± 6.94 years, BH = 182.60 ± 6.61 cm, BM = 86.44 ±11.25 kg, and BMI = 25.97 ± 3.21 kg/m^2^); and general police (age = 32.96 ± 5.59 years, BH = 182.44 ± 8.59 cm, BM = 92.24 ± 12.36 kg, and BMI = 27.66 ± 2.37 kg/m^2^). All participants were informed about the purpose of the study and were assessed after providing informed consent. Data for this study were collected in a de-identified format without obtaining names or any personal identification. To match the different types of data collected, participants were coded using an alpha numeric coding system (e.g., for a police officer the designator PO was used in addition to a sequential number (i.e., PO1-PO237). The ethical board of the Faculty of Sport and Physical Education, University of Belgrade, Serbia provided the ethical approval (no. 484-2) for this study. The study was conducted in accordance with the Declaration of Helsinki [26].

### 2.2. Occupational Characteristics

The organizational structure of the police of the Republic of Serbia is based on the responsibilities covered by the Ministry of Interior and occupational processes within the Ministry [27]. Organizational units are hierarchically structured, sorted and interconnected, aiming to provide timely but appropriate responses to safety and security threats to the public. Therefore, the tasks, specializations, responsibilities, and jurisdictions of every police unit are well defined, albeit with some being more and others less physically demanding.

The SAU is the most elite police tactical unit in Serbia specializing in weapons and tactics, preventing complex anti-terrorist tasks, hostage crises interdiction, VIP protection, high-risk arrests, and other complex security tasks [28]. The physical employment standards for SAU are very rigorous and only a limited number of candidates are accepted. Their physical fitness is required to be at very high standards at all times, with these standards regularly confirmed by general and specific physical fitness assessments. Therefore, these personnel are required to regularly participate in strength and conditioning programs along with specialized training, involving weapons and tactics, which in itself is often physically demanding.

Gendarmerie of the Republic of Serbia is another police tactical unit who specialize in crowd and riot control, search and rescue, weapons and tactics training, border control, some general police duties, and support of the SAU in case of terrorist attack [29]. They maintain public order during high-risk gatherings, such as protests, riots or sporting events, where there are potentially larger numbers of hooligans. Their recruitment and training is rigorous and physical fitness is regularly checked, although less so than in the SAU. However, they must be physically prepared to respond to a given situation at short notice, which is why they are expected to follow specific and general strength and conditioning programs.

Firefighters of the Republic of Serbia are the part of the civil defence specialized for fire and rescue operations and operating in case of man-caused or natural disasters. Their physical fitness at recruitment and during their career needs to be high, given that tasks conducted during operations are often very physically demanding. Moreover, the protective clothing, self-contained breathing apparatus, and other tools needed for certain tasks constitute a heavy external load which they are required to wear and carry. Therefore, physical fitness and specific skills are assessed on a regular basis and in order to attain the expected levels, they may need to implement general and specific strength and conditioning programs [30,31].

Police officers of general jurisdiction perform preventive and operative tasks aimed to establish public security and safety. Furthermore, they patrol the streets and inter-city roads, conduct investigative tasks, complete administrative work, and respond to reported offences, homicides, traffic accidents, and more. Although physical fitness assessment is part of the recruitment and initial training at the academy, once they enter the force, it is not as rigorously or frequently checked as in the tactical units such as SAU and Gendarmerie.

### 2.3. Body Composition Assessment

A multichannel bioelectric impendence analyser InBody 720 (IBM, Seoul, Korea) was used for the assessment of body composition. This device has been found to be valid and reliable [32,33]. The assessment was conducted according to previously reported procedures [5,16]. In short, participants visited the Laboratory of the Faculty of Sport and Physical Education, University of Belgrade during early hours of the day, 08:00–10:00 a.m. They were assessed in underwear and barefoot, with their feet positioned on designated spots and their hands grasping handles by positioning their fingers on designated spots. They stood straight, looking forward, with hands extended next to their body, slightly abducted (i.e., not touching the sides of their upper body). Participants were informed about the testing through the agency. Ahead of time, they were informed about the timing of the assessment and asked to abstain from strenuous exercise 24 h prior to testing, eating a large meal the night before (i.e., for dinner), and to avoid having breakfast and fluids on the morning of the test. However, they were also informed that the testing would take approximately 5–10 min and that they would be provided with water and a snack bar after the assessment. Body composition results were recorded by the device’s software and printed out as a results sheet. Besides age, BH and BM, participant BMI, %BF, %SMM were also provided, while the index of hypokinesia (IH) was calculated as %BF divided by BMI [5].

### 2.4. Frequency and Volume of Leisure Time Physical Activity

Immediately after they completed the body composition assessment, participants were provided with an international physical activity questionnaire (IPAQ) that assessed their levels of physical activity [34]. The questionnaire consists of four sections—household physical activity, transportation physical activity, physical activity at work, and aerobic leisure time physical activity—and it provides valid information for physical activity levels [34]. The number of days per week and the time per day spent in walking, moderate or vigorous activity were collected for each domain, but only the LTPA findings were analyzed. The days were multiplied with hours for each of the three sets of questions (walking, moderate, vigorous), and then those three numbers were summed to get a total volume. Participants were divided into groups based on the data that they provided on their volume of LTPA (V-LTPA; i.e., total number of minutes spent doing physical activity within one week). Three groups were formed according to the recommended weekly physical activity [35,36]: sedentary = 0–149 min per week, moderately active = 150–300 min per week and very active = more than 300 min per week.

### 2.5. Statistical Analysis

All data were collected and entered into Microsoft Excel and transferred to the Statistical Package for Social Sciences (IBM, SPSS Statistics 20, Armonk, NY, USA) for statistical analyses. Descriptive statistics for mean, standard deviation, minimum and maximum values were calculated for each occupation group relative to V-LTPA. Pearson’s correlation analysis was conducted to establish associations between age, occupation, V-LTPA, BMI, %BF, %SMM, and IH. For this purpose, occupations were coded as: 1—SAU, 2—Gendarmerie, 3—firefighters, and 4—general police, with 1 being the most demanding and 4 being the least physically demanding occupation. The magnitude of the effects was defined as follows: small = 0.2, moderate = 0.6, large = 1.2 and very large = 2.0 [37]. Multivariate analysis of variance (4 × 3) followed by Bonferroni post-hoc analysis was used to investigate the between-occupation differences in body composition and V-LTPA (first hypothesis) as well as the effect of V-LTPA within-occupation, and effect of V-LTPA between occupations on body composition (second hypothesis). The significance level was set to *p* < 0.05. Cohen’s effect sizes (*d*) were calculated as the ratio of the difference in mean scores to standard deviation, following the formula: ES = (M_2_ − M_1_)/SD, where M_1_ and M_2_ were the means of the groups investigated and the SD was a pooled standard deviation of compared groups. Relative differences (%) and *d* showed the sizes of differences obtained in the aforementioned comparisons.

## 3. Results

Descriptive statistics for age, BH, BMI, %BF, %SMM, and IH for each occupation group and relative to LTPA level are shown in Table 1. The percentage of sedentary officers was 14.8% for SAU (*n* = 8), 47.3% for Gendarmerie (*n* = 26), 76.5% for firefighters (*n* = 52), and 73.3% for general police (*n* = 44). Moderately active officers comprised 42.6% of SAU (*n* = 23), 29.1% of Gendarmerie (*n* = 16), 14.7% of firefighters (*n* = 10), and 21.7% of general police (*n* = 13). Finally, the percentage of very active officers was 42.6% for SAU (*n* = 23), 23.6% for Gendarmerie (*n* = 13), 8.8% for firefighters (*n* = 6), and 5% for general police (*n* = 3).

Correlation analysis revealed significant negative associations (*p* < 0.01) of V-LTPA with age and occupation, while age, occupation and V-LTPA were negatively correlated with BMI, %BF, and IH, and positively with %SMM (Table 2). Correlations were the strongest between V-LTPA and IH (r = −0.72), followed by %SMM and %BF (r = 0.70).

Analysis of variance revealed significant differences between occupation groups in %BF (F = 11.64, *p* < 0.001), %SMM (F = 13.58, *p* < 0.001), IH (F = 14.30, *p* < 0.001), and V-LTPA (F = 32.51, *p* < 0.001), but not in age (F = 1.17, *p* = 0.322) or BMI (F = 2.14, *p* = 0.096). Post hoc analysis (Table 3) revealed that SAU officers had lower %BF and IH, and higher %SMM than the officers from all other police occupations. Furthermore, Gendarmerie had lower %BF and IH and higher %SMM than general police, while firefighters only had lower IH than general police. SAU officers also reported higher V-LTPA than Gendarmerie, firefighters and general. Gendarmerie officers reported higher V-LTPA than firefighters and general police, while no difference occurred between firefighters and general police.

**Figure 1:**
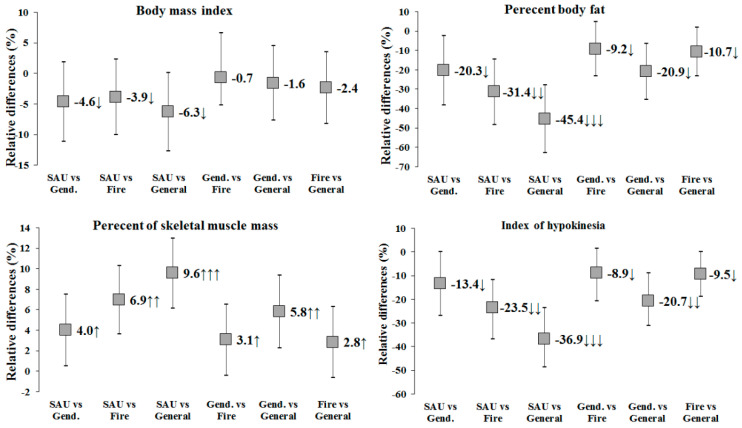
Relative (%) between-occupation differences and magnitude of differences. Small (↓), moderate (↓↓) and large (↓↓↓) negative difference. Small (↑), moderate (↑↑), and large (↑↑↑) positive difference.

Although differences in BMI were not significant, a lower BMI of small magnitude could be observed in SAU officers compared to firefighters and general police (Figure 1). However, differences in body composition between occupations were small to large, with more physically demanding occupations having lower %BF and IH levels and higher %SMM.

Considering the within-occupation differences relative to V-LTPA, as reported, where physical activity level was higher, the BMI, %BF, and IH were lower, while %SMM was higher (Table 4). Among SAU officers, BMI was significantly lower in moderately active than in very active officers. There was no difference in BMI by V-LTPA among Gendarmerie officers. Moderately active firefighters and general police had significantly lower BMIs than those who were sedentary in each group. A significant difference in BMI was found between very active and sedentary firefighters. %BF and IH were lower in very active officers than in sedentary officers in all occupations, while significant differences were found in %BF and IH between moderately active and sedentary Gendarmerie, firefighters, and general police. Significantly lower %BF and IH were only found for very active compared to moderately active SAU officers. Finally, %SMM was higher in moderately active than in sedentary firefighters and general police, and in very active than in sedentary SAU, firefighters and general police. Only very active SAU officers had a significantly higher %SMM than moderately active SAU officers.

**Figure 2:**
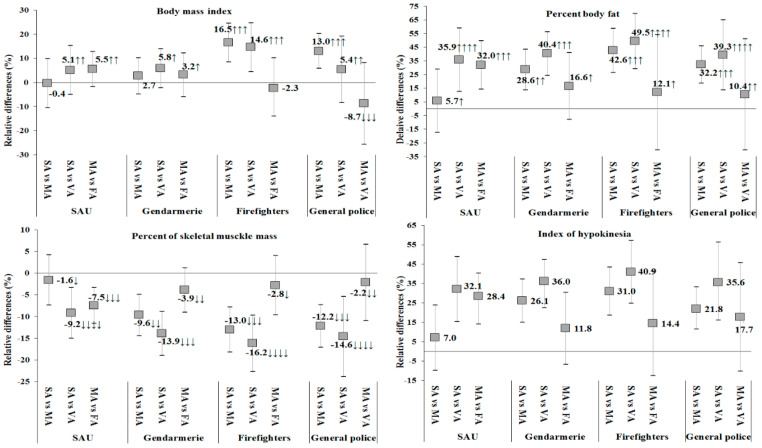
Relative differences (%) in body composition within each occupational group relative to V-LTPA. Small (↓), moderate (↓↓), large (↓↓↓) and very large (↓↓↓↓) negative difference. Small (↑), moderate (↑↑), large (↑↑↑), and very large (↑↑↑↑) positive difference. SA—Sedentary, MA—Moderately active, VA—Very active.

Examination of the magnitudes of within-group differences (Figure 2) by occupation and V-LTPA found moderate, small, large and very large differences in BMI for SAU, Gendarmerie, firefighters, and general officers, respectively. Differences in %BF and %SMM were small to large in Gendarmerie and small to very large in SAU, firefighters, and general police. Differences in IH were small to large in SAU and Gendarmerie, and small to very large in firefighters and general officers.

The differences in body composition between officers of the same V-LTPA but different occupation are presented in Table 5. Moderately active firefighters had a significantly lower BMI than both SAU officers and Gendarmerie officers. Among sedentary officers, the %BF of SAU officers was lower than that of general police, while among moderately active officers, firefighters had lower %BF than SAU. Furthermore, the %SMM of sedentary officers was higher in SAU compared to firefighters and general police, while the IH of sedentary general police was higher than SAU and firefighters. Similar to %BF, the IH of Firefighters was lower than that of SAU officers. There was no significant difference in the body composition of very active police officers, regardless of their occupation.

## 4. Discussion

This study investigated differences in the body composition and levels of LTPA between various police occupations, including whether LTPA moderated the relationship between occupation and body composition. As hypothesized, we found that officers with more physically demanding occupations had moderately better body composition, as well as a higher V-LTPA. This was further reflected in lower relative body fatness and higher relative muscularity of officers from more physically demanding duties compared to those whose duties were less demanding. To that end, Boyce et al. [38] found that firefighters had lower %BF than officers from a police department, while Dawes et al. [39] found that, even among SWAT officers, those who are not required to perform physical training while on duty (i.e., part-time SWAT officers) had higher BMI and body fatness. Based on these findings it appears that body composition may be influenced by both LPTA and specific occupational specialty.

Officers from more physically demanding occupations reported greater physical activity during leisure time, which may help to explain the differences in body composition between groups. Considering this, analysis of within-group differences revealed that the reported V-LTPA had small to very large effects on all investigated indicators of body composition, with more physically active officers being leaner, regardless of their occupation. This is in accordance with a study that investigated the differences in body composition of general police officers with respect to reported level of LTPA, where those who reported a higher frequency and volume of LTPA had a lower %SMM and BFMI, while having higher %SMM, and SMMI [16]. Dominski et al. [40] found significant negative associations between the level of physical activity and %BF and BMI in a sample of Brazilian police officers. Moreover, research conducted on a police academy showed that BMI and waist circumference significantly decreased while police cadets were at the academy (i.e., during semester) and increased during a 2-month semester break when they were less physically active [41]. Thus, a higher V-LTPA has been consistently associated with better body composition among police officers, regardless of the nature of their duties.

We adjusted the between-group analysis to the level of V-LTPA (between-within design), but found no significant difference between very active officers from different occupations. Thus, as hypothesized, physical activity moderated the relationship between occupation and body composition. However, physical activity only partially moderated the relationship since, among moderately active participants, SAU had significantly lower body fat and IH than firefighters, while in sedentary participants, SAU had significantly lower body fat and IH and higher muscle mass than general police. Accordingly, our results suggest that as the V-LTPA reduces, occupational demands may play a larger role in body composition. The lower body fatness of sedentary SAU officers may lie in the recruitment and service (i.e., employment) requirements as well as more frequent fitness assessments that officers must meet in order to remain in service. This demonstrates importance, as studies suggest that police officer LTPA tends to decrease, while BMI and body fatness tend to increase, with the time spent in service [12,15,42,43]. Obesity has been found across police populations [44,45,46], whereby the effort to battle this issue has been based mainly on the implementation of exercise programs [6,17,18]. In consideration of our results, encouraging officers to increase their V-LTPA may be an effective way to help moderate officers’ body composition across various police occupations.

## 5. Study limitations

Due to the cross-sectional nature of this study, we cannot determine whether time in occupation resulted in the significant differences found or whether more fit and physically active officers with better body composition were recruited into more demanding occupations in the first place. This study lacked an objective prospective measure of physical activity and did not account for differences in physical fitness training on the job versus in personal leisure time. The results of this study may differ from those found in other countries and additional research should be conducted with female officers. In addition, information on family tasks and their association with age could be considered in the future, and whether older police officers were also higher ranked in police organizations so their duties were more organizational and supervision-based rather than in the field.

## 6. Conclusions

This was the first study to investigate the differences in body composition and LTPA across several police occupations and whether LTPA moderated that relationship. Our results suggest that both occupation and V-LTPA were significantly related to the body composition of police officers, with officers from more physically demanding occupations with higher V-LTPA demonstrating better body composition. However, V-LTPA partially moderated the relationship between occupation and body composition, thereby showing its viability as a potential intervention target. This relationship seemed to have a protective effect, as body composition was similar between police officers from different occupations if they reported to be physically very active. Thereby, higher volumes of LTPA (i.e., 300+ min/week) seem to be sufficient for a good body composition of police officers, as none of the participants from this group were obese (i.e., had %BF above 25%). Moreover, moderately active officers had lower %BF and IH (i.e., better balance between body fatness and muscularity) than those who reported to be sedentary. Therefore, officers should aim to be at least moderately physically active during leisure time (i.e., 150–300 min/week) for some improvements, while, for greater improvements, they may need to aim for a higher volume of activity.

## Figures and Tables

**Table 1:** Descriptive statistics for study participants (N = 237).

Var.	Group	Sedentary		Moderately Active		Very Active	
		Mean ± SD	Min.–Max.	Mean ± SD	Min.–Max.	Mean ± SD	Min.–Max.
Age (years)	SAU	40.13 ± 5.77	32.00–49.00	36.41 ± 3.68	28.00 ± 43.00	33.63 ± 5.45	24.00 ± 44.00
	Gendarmerie	41.0 ± 35.44	31.40–50.00	33.77 ± 5.42	25.00 ± 47.00	32.15 ± 3.77	28.00 ± 41.00
	Firefighters	38.60 ± 7.40	24.00–52.00	32.90 ± 6.72	21.00 ± 46.00	31.83 ± 6.71	23.00 ± 43.00
	General police	36.00 ± 6.66	25.00–52.00	33.94 ± 6.21	24.30 ± 46.60	28.93 ± 3.89	26.30 ± 33.40
BH (cm)	SAU	181.40 ± 3.75	177.40–188.30	180.57 ± 5.41	171.70 ± 194.50	182.91 ± 6.74	166.10 ± 196.60
	Gendarmerie	180.52 ± 5.76	168.50–190.30	183.91 ± 8.45	173.50 ± 197.60	180.93 ± 6.94	171.70 ± 192.80
	Firefighters	179.51 ± 6.35	162.70–191.70	185.92 ± 5.88	172.60 ± 193.40	182.38 ± 7.60	174.80 ± 193.40
	General police	182.88 ± 7.18	167.80–206.20	183.28 ± 7.83	171.50 ± 202.40	181.17 ± 10.77	170.80 ± 192.30
BM (kg)	SAU	90.34 ± 10.78	78.80–106.40	89.76 ± 8.30	75.40 ± 106.00	87.30 ± 9.64	66.00 ± 109.40
	Gendarmerie	93.74 ± 15.92	71.00–129.50	94.91 ± 18.92	72.70 ± 146.30	89.09 ± 12.69	72.40 ± 112.80
	Firefighters	93.48 ± 13.74	66.60–125.30	83.39 ± 9.21	67.90 ± 93.20	82.45 ± 10.80	63.00 ± 92.70
	General police	98.98 ± 17.08	70.20–145.90	86.18 ± 10.18	62.60 ± 104.70	91.57 ± 9.81	84.20 ± 102.70
BMI (kg/m^2^)	SAU	27.43 ± 2.81	23.12–31.70	27.52 ± 2.19	22.94 ± 31.59	26.02 ± 1.51	23.86 ± 29.68
	Gendarmerie	28.73 ± 4.45	21.38–38.80	27.96 ± 4.82	23.07 ± 44.26	27.06 ± 1.94	23.71 ± 30.35
	Firefighters	28.98 ± 3.81	20.91–39.21	24.20 ± 3.25	20.53 ± 31.02	24.75 ± 2.57	19.91 ± 26.80
	General police	29.47 ± 3.83	23.37–40.42	25.63 ± 2.32	19.43 ± 29.00	27.87 ± 0.95	26.98 ± 28.86
%BF (%)	SAU	19.59 ± 3.64	13.45–24.21	18.46 ± 4.02	5.31 ± 26.51	12.56 ± 3.32	6.87 ± 18.12
	Gendarmerie	23.61 ± 7.80	6.48–36.95	16.85 ± 5.79	12.10 ± 36.02	14.07 ± 4.32	7.98 ± 22.65
	Firefighters	23.69 ± 6.45	2.96–38.97	13.60 ± 6.11	5.47 ± 26.08	11.97 ± 3.76	5.87 ± 16.06
	General police	25.74 ± 5.85	14.97–40.20	17.45 ± 4.00	10.06 ± 22.73	15.63 ± 1.60	14.51 ± 17.46
%SMM (%)	SAU	45.95 ± 1.83	43.70–48.86	46.68 ± 2.40	41.91 ± 54.51	50.17 ± 2.12	46.49 ± 54.03
	Gendarmerie	43.48 ± 4.40	35.86–52.68	47.68 ± 3.28	36.84 ± 50.43	49.53 ± 2.59	44.61 ± 53.72
	Firefighters	43.28 ± 4.01	31.37–55.47	48.91 ± 3.67	41.77 ± 54.44	50.28 ± 1.59	47.69 ± 52.22
	General police	42.04 ± 3.22	33.53–48.87	47.18 ± 2.37	44.16 ± 51.37	48.19 ± 0.91	47.15 ± 48.75
IH (Index)	SAU	0.72 ± 0.13	0.58–0.87	0.67 ± 0.13	0.23 ± 0.89	0.48 ± 0.13	0.27 ± 0.75
	Gendarmerie	0.80 ± 0.18	0.28–1.06	0.59 ± 0.10	0.45 ± 0.81	0.52 ± 0.15	0.26 ± 0.79
	Firefighters	0.81 ± 0.16	0.14–1.07	0.56 ± 0.21	0.22 ± 0.84	0.48 ± 0.13	0.30 ± 0.66
	General police	0.87 ± 0.15	0.57–1.17	0.68 ± 0.13	0.42 ± 0.87	0.56 ± 0.04	0.52 ± 0.60

SD—standard deviation, BH—body height, BM—body mass, BMI—body mass index, %BF—percent of body fat, %SMM—percent of skeletal muscle mass, IH—index of hypokinesia.

**Table 2:** Correlation analysis.

Variables	Age	Occupation	V-LTPA
Age	-	-	-
Occupation	−0.047	-	-
V-LTPA	−0.331 **	−0.475 **	-
BMI	0.183 **	0.140 *	−0.355 **
%BF	0.334 **	0.348 **	−0.687 **
%SMM	−0.345 **	−0.371 **	0.698 **
IH	0.352 **	0.386 **	−0.721 **

** Significant at *p* < 0.01, * Significant at the *p* < 0.05. V-LTPA—volume of leisure time physical activity.

**Table 3:** Differences between police occupations in body composition and leisure-time physical activity obtained by Bonferroni post hoc tests.

Between-Occupation Comparison		%BF	%SMM	IH (Index Unit)	V-LTPA (min/week)
		Mean 95% CI	Mean 95% CI	Mean 95% CI	Mean 95% CI
SAU	Gendarmerie	−3.27 * −6.14–0.40	1.92 * 0.243.60	−0.08 * −0.16–0.00	97.17 * 32.11–162.23
	Firefighters	−5.06 * −7.79–2.33	3.33 * 1.73–4.93	−0.14 * −0.22–0.07	195.18 * 133.28–257.08
	General police	−7.32 * −10.13–4.51	4.59 * 2.95–6.24	−0.22 * −0.29–0.14	203.93 * 140.23–267.63
Gendarmerie	Firefighters	−1.79 −4.51–0.93	1.41 −0.18–3.00	−0.06 −0.14–0.01	98.01 * 36.42–159.59
	General police	−4.05 * −6.85–1.25	2.67 * 1.03–4.31	−0.14 * −0.21–0.06	106.76 * 43.36–170.16
Firefighters	General police	−2.26 −4.92–0.39	1.26 −0.29–2.81	−0.07 * −0.14–0.00	8.75 −51.40–68.90

* Significant at *p* < 0.05. %BF—percent of body fat, %SMM—percent of skeletal muscle mass, IH—index of hypokinesia, V-LTPA—volume of leisure time physical activity, CI—confidence interval.

**Table 4:** Within-occupation differences in body composition relative to leisure-time physical activity obtained by Bonferroni post hoc tests.

Variables	Within-Between Comparison		SAU	Gendarmerie	Firefighters	General Police
			Mean 95% CI	Mean 95% CI	Mean 95% CI	Mean 95% CI
BMI (kg/m^2^)	ANOVA		F = 3.51, *p* = 0.037	F = 0.72, *p* = 0.492	F = 9.62, *p* < 0.001	F = 6.11, *p* = 0.004
	Sedentary	Moderately active	−0.10 −2.88–2.69	0.78 −1.38–2.93	4.78 * 2.44–7.13	3.84 * 1.70–5.98
		Very active	1.41 −1.38–4.19	1.67 −0.64–3.97	4.23 * 1.30–7.15	1.60 −2.45–5.65
	Moderately active	Very active	1.51 * −0.49–3.51	0.89 −1.64–3.42	−0.55 −4.06–2.95	−2.24 −6.58–2.11
%BF (%)	ANOVA		F = 18.99, *p* < 0.001	F = 10.81, *p* < 0.001	F = 18.14, *p* < 0.001	F = 15.09, *p* < 0.001
	Sedentary	Moderately active	1.12 −3.43–5.68	6.76 * 3.23–10.28	10.09 * 6.26–13.93	8.30 * 4.79–11.80
		Very active	7.03 * 2.47–11.58	9.54 * 5.77–13.31	11.73 * 6.94–16.51	10.11 * 3.49–16.73
	Moderately active	Very active	5.90 * 2.63–9.18	2.79 −1.35–6.93	1.64 −4.10–7.37	1.82 −5.29–8.93
%SMM (%)	ANOVA		F = 18.59, *p* < 0.001	F = 13.28, *p* < 0.001	F = 18.14, *p* < 0.001	F = 18.51, *p* < 0.001
	Sedentary	Moderately active	−0.73 −3.40–1.93	−4.19 −6.26–2.13	−5.64 * −7.88–3.39	−5.13 * −7.18–3.08
		Very active	−4.22 * −6.89–1.55	−6.05 −8.26–3.84	−7.00 * −9.81–4.20	−6.15 * −10.03–2.27
	Moderately active	Very active	−3.49 * −5.40–1.57	−1.86 −4.28–0.57	−1.37 −4.72–1.99	−1.02 −5.18–3.14
IH (Index unit)	ANOVA		F = 15.77, *p* < 0.001	F = 17.47, *p* < 0.001	F = 18.27, *p* < 0.001	F = 14.73, *p* < 0.001
	Sedentary	Moderately active	0.05 −0.07–0.17	0.21 * 0.12–0.30	0.25 * 0.15–0.35	0.19 * 0.10–0.29
		Very active	0.23 * 0.11–0.35	0.29 * 0.18–0.38	0.33 * 0.20–0.46	0.31 * 0.14–0.49
	Moderately active	Very active	0.19 * 0.10–0.27	0.07 −0.04–0.18	0.08 −0.07–0.23	0.12 −0.07–0.31

* Significant at *p* < 0.05. %BF—percent of body fat, %SMM—percent of skeletal muscle mass, IH—index of hypokinesia, V-LTPA—volume of leisure time physical activity, CI—confidence interval.

**Table 5:** Between-occupation differences in body composition relative to leisure-time physical activity obtained by Bonferroni post hoc tests.

Variables	Between-Within Comparison		Sedentary	Moderately Active	Very Active
			Mean 95% CI	Mean 95% CI	Mean 95% CI
BMI (kg/m^2^)	SAU	Gendarmerie	−1.31 −4.05–1.44	−0.43 −2.64–1.78	−1.05 −3.40–1.31
		Firefighters	−1.55 −4.13–1.02	3.33 * 0.76–5.90	1.27 −1.84–4.38
		General police	−2.05 −4.65–0.56	1.89 −0.46–4.25	−1.86 −6.02–2.31
	Gendarmerie	Firefighters	−0.25 −1.88–1.38	3.76 * 1.02–6.49	2.32 −1.03–5.66
		General police	−0.74 −2.42–0.94	2.32 −0.021–4.86	−0.81 −5.15–3.54
	Firefighters	General police	−0.49 −1.88–0.90	−1.44 −4.29–1.42	−3.12 −7.92–1.68
%BF (%)	SAU	Gendarmerie	−4.02 −8.51–0.47	1.61 −2.00–5.22	−1.50 −5.36–2.35
		Firefighters	−4.11 −8.32–0.11	4.86 * 0.66–9.07	0.59 −4.49–5.68
		General police	−6.15 * −10.42–1.89	1.02 −2.83–4.87	−3.07 −9.88–3.74
	Gendarmerie	Firefighters	−0.09 −2.75–2.58	3.25 −1.22–7.73	2.10 −3.38–7.58
		General police	−2.13 −4.88–0.61	−0.59 −4.74–3.55	−1.56 −8.67–5.54
	Firefighters	General police	−2.05 −4.32–0.23	−3.85 −8.51–0.82	−3.66 −11.51–4.18
%SMM (%)	SAU	Gendarmerie	2.46 −0.16–5.09	−1.00 −3.11–1.12	0.63 −1.62–2.89
		Firefighters	2.67 * 0.20–5.14	−2.23 −4.69–0.23	−0.11 −3.09–2.86
		General police	3.90 * 1.41–6.40	−0.50 −2.75–1.76	1.97 −2.02–5.96
	Gendarmerie	Firefighters	0.21 −1.35–1.77	−1.24 −3.86–1.38	−0.75 −3.95–2.46
		General police	1.44 −0.17–3.05	0.50 −1.93–2.92	1.34 −2.82–5.50
	Firefighters	General police	1.23 −0.10–2.56	1.74 −1.00–4.47	2.09 −2.51–6.68
IH (Index unit)	SAU	Gendarmerie	−0.09 −0.21–0.03	0.08 −0.02–0.17	−0.04 −0.14–0.07
		Firefighters	−0.09 −0.20–0.02	0.11 * 0.00–0.22	0.01 −0.13–0.14
		General police	−0.15 * −0.27–0.04	−0.01 −0.11–0.09	−0.08 −0.26–0.010
	Gendarmerie	Firefighters	0.00 −0.07–0.07	0.04 −0.08–0.15	0.04 −0.10–0.19
		General police	−0.07 −0.14–0.01	−0.08 −0.19–0.03	−0.04 −0.23–0.15
	Firefighters	General police	−0.06 * −0.12–0.00	−0.12 −0.24–0.00	−0.08 −0.29–0.12

* Significant at *p* < 0.05. %BF—percent of body fat, %SMM—percent of skeletal muscle mass, IH—index of hypokinesia, V-LTPA—volume of leisure time physical activity, CI—confidence interval.

## References

[1] Garbarino S., Magnavita N. (2015). Work stress and metabolic syndrome in police officers. A prospective study. PLoS ONE.

[2] Hauschild V.D., DeGroot D.W., Hall S.M., Grier T.L., Deaver K.D., Hauret K.G., Jones B.H. (2017). Fitness tests and occupational tasks of military interest: A systematic review of correlations. Occup. Environ. Med..

[3] Maupin D., Wills T., Orr R., Schram B. (2018). Fitness profiles in elite tactical units: A critical review. Int. J. Exerc. Sci..

[4] Riebe D., Ehrman J.K., Liguori G., Megal M. (2018). ACSM’s Guidelines for Exercise Testing and Prescription.

[5] Kukic F., Dopsaj M., Dawes J., Orr R., Cvorovic A. (2018). Use of human body morphology as an indicator of physical fitness: Implications for police officers. Int. J. Morphol..

[6] Kukić F., Čvorović A. (2019). The strategic approach to an improvement of health-related physical fitness of police officers: An 8-week exercise intervention: Pilot study. Bezb. Beogr..

[7] Guffey J.E., Larson J.G., Lasley J. (2015). Police officer fitness, diet, lifestyle and its relationship to duty performance and injury. J. Legal Issues Cases Bus..

[8] Dawes J.J., Lindsay K., Bero J., Elder C., Kornhauser C., Holmes R. (2017). Physical fitness characteristics of high vs. low performers on an occupationally specific physical agility test for patrol officers. J. Strength Cond. Res..

[9] Mona G.G., Chimbari M.J., Hongoro C. (2019). A systematic review on occupational hazards, injuries and diseases among police officers worldwide: Policy implications for the South African Police Service. J. Occup. Med. Toxicol..

[10] Orr R., Pope R., Stierli M., Hinton B. (2017). Grip strength and its relationship to police recruit task performance and injury risk: A retrospective cohort study. Int. J. Environ. Res. Public Health.

[11] Dawes J.J., Orr R.M., Siekaniec C.L., Vanderwoude A.A., Pope R. (2016). Associations between anthropometric characteristics and physical performance in male law enforcement officers: A retrospective cohort study. Ann. Occup. Environ. Med..

[12] Sorensen L., Smolander J., Louhevaara V., Korhonen O., Oja P. (2000). Physical activity, fitness and body composition of Finnish police officers: A 15-year follow-up study. Occup. Med..

[13] Charles L.E., Burchfiel C.M., Violanti J.M., Fekedulegn D., Slaven J.E., Browne R.W., Hartley T.A., Andrew M.E. (2008). Adiposity measures and oxidative stress among police officers. Obesity.

[14] Gu J.K., Charles L.E., Burchfiel C.M., Fekedulegn D., Sarkisian K., Andrew M.E., Ma C., Violanti J.M. (2012). Long work hours and adiposity among police officers in a US Northeast City. J. Occup. Environ. Med..

[15] Ćopić N., Kukić F., Tomić I., Parčin I., Dopsaj M. (2020). The impact of shift-work on nutritional status of police officers. J. Crim. Law.

[16] Vuković M., Kukić F., Čvorović A., Janković D., Prćić I., Dopsaj M. (2020). Relations between frequency and volume of leisure-time physical activity and body composition in police officers. Res. Q. Exerc. Sport.

[17] Čvorović A., Kukić F., Orr R.M., Dawes J.J., Jeknić V., Stojković M. (2018). Impact of a 12-week postgraduate training course on the body composition and physical abilities of police trainees. J. Strength Cond. Res..

[18] Demling R.H., DeSanti L. (2000). Effect of a hypocaloric diet, increased protein intake and resistance training on lean mass gains and fat mass loss in overweight police officers. Ann. Nutr. Metab..

[19] Violanti J.M., Hartley T.A., Gu J.K., Fekedulegn D., Andrew M.E., Burchfiel C.M. (2013). Life Expectancy in Police Officers: A Comparison with the U.S. General Population. Int. J. Emerg. Ment. Health.

[20] Acquadro Maran D., Zedda M., Varetto A. (2018). Organizational and occupational stressors, their consequences and coping strategies: A questionnaire survey among Italian patrol police officers. Int. J. Environ. Res. Public Health.

[21] Ma C.C., Burchfiel C.M., Fekedulegn D., Andrew M.E., Charles L.E., Gu J.K., Mnatsakanova A., Violanti J.M. (2011). Association of shift work with physical activity among police officers: The Buffalo cardio-metabolic occupational police stress study. J. Occup. Environ. Med..

[22] Pryor R.R., Colburn D., Crill M.T., Hostler D.P., Suyama J. (2012). Fitness characteristics of a suburban special weapons and tactics team. J. Strength Cond. Res..

[23] Smith D.L. (2011). Firefighter Fitness: Improving Performance and Preventing Injuries and Fatalities. Curr. Sports Med. Rep..

[24] Clark J.G., Jackson M.S., Schaefer P.M., Sharpe E.G. (2000). Training SWAT teams: Implications for improving tactical units. J. Crim. Justice.

[25] MacDonald D., Pope R., Orr R. (2016). Differences in Physical Characteristics and Performance Measures of Part-Time and Full- Time Tactical Personnel: A Critical Narrative Review. J. Mil. Veterans Health.

[26] Williams J.R. (2008). The declaration of Helsinki and public health. Bull World Health Organ..

[27] Subošić D. (2020). Organizacija i Poslovi Policije.

[28] Specijalne-jedinice.com | Specijalna antiteroristička jedinica-SAJ. https://specijalne-jedinice.com/Srbija/SAJ.html#sthash.jKwlw7Y2.yXqNOWAI.dpbs.

[29] Specijalne-jedinice.com | Gendarmerie of the Republic of Serbia. https://specijalne-jedinice.com/Srbija/Zandarmerija-English.html#sthash.5p7ruaFn.ILhj4BeS.dpbs.

[30] Abel M.G., Sell K., Dennison K. (2011). Design and Implementation of Fitness Programs for Firefighters. Strength Cond. J..

[31] Cvorovic A., Kukić F., Abdulovic A., Orr R.M., Dawes J. (2020). Effectiveness of a short-term conditioning program to prepare firefighters for an occupationally-specific competition-pilot study. J. Aust. Strength Cond..

[32] Aandstad A., Holtberget K., Hageberg R., Holme I., Anderssen S.A. (2014). Validity and reliability of bioelectrical impedance analysis and skinfold thickness in predicting body fat in military personnel. Mil. Med..

[33] Kim M., Shinkai S., Murayama H., Mori S. (2015). Comparison of segmental multifrequency bioelectrical impedance analysis with dual-energy X-ray absorptiometry for the assessment of body composition in a community-dwelling older population. Geriatr. Gerontol. Int..

[34] Craig C.L., Marshall A.L., Sjöström M., Bauman A.E., Booth M.L., Ainsworth B.E., Pratt M., Ekelund U., Yngve A., Sallis J.F. (2003). International physical activity questionnaire: 12-country reliability and validity. Med. Sci. Sports Exerc..

[35] Powell K.E., Paluch A.E., Blair S.N. (2011). Physical activity for health: What kind? How much? How intense? On top of what?. Annu. Rev. Public Health.

[36] Blair S.N., LaMonte M.J., Nichaman M.Z. (2004). The evolution of physical activity recommendations: How much is enough?. Am. J. Clin. Nutr..

[37] Sullivan G.M., Feinn R. (2012). Using effect size—or why the *P* value is not enough. J. Grad. Med Educ..

[38] Boyce R.W., Ciulla S., Jones G.R., Boone E.L., Elliott S.M., Combs C.S. (2008). Muscular Strength and Body Composition Comparison Between the Charlotte-Mecklenburg Fire and Police Departments. Int. J. Exerc. Sci..

[39] Dawes J.J., Orr R.M., Elder C.L., Rockwell C. (2014). Association between fatness and measures of muscular endurance among part-time SWAT officers. J. Aust. Strength Cond..

[40] Dominski F.H., Crocetta T.B., Santo L.B.D.E., Cardoso T.E., da Silva R., Andrade  A. (2018). Police Officers Who Are Physically Active and Have Low Levels of Body Fat Show Better Reaction Time. J. Occup. Environ. Med..

[41] Kukić F., Jeknić V., Dawes J., Orr R., Stojković M., Čvorović A. (2019). Effects of training and a semester break on physical fitness of police trainees. Kinesiology.

[42] Boyce R.W., Jones G., Lloyd C. (2008). A longitudinal observation of police: Body composition changes over 12 years with gender and race comparisons. J. Exerc. Physiol. Online.

[43] Lagestad P., van den Tillaar R. (2014). Longitudinal changes in the physical activity patterns of police officers. Int. J. Police Sci. Manag..

[44] Heinrich K.M., Gurevich K.G., Arkhangelskaia A.N., Karazhelyaskov O.P., Poston W.S.C. (2020). Despite low obesity rates, body mass index under-estimated obesity among Russian police officers when compared to body fat percentage. Int. J. Environ. Res. Public Health.

[45] Gurevich K.G., Poston W.S.C., Anders B., Ivkina M.A., Archangelskaya A., Jitnarin N., Starodubov V.I. (2017). Obesity prevalence and accuracy of BMI-defined obesity in Russian firefighters. Occup. Med. (Lond.).

[46] Dopsaj M., Vuković M. (2015). Prevalence of the body mass index (BMI) among the members of the Ministry of Interior of the Republic of Serbia: Pilot study. Bezb. Beogr..

